# CYFRA 21-1 in patients with suspected cancer: evaluation of an optimal cutoff to assess the diagnostic efficacy and prognostic value

**DOI:** 10.1515/almed-2020-0005

**Published:** 2020-10-08

**Authors:** Sonsoles Garcia-Valdecasas Gayo, Maria Jesus Ruiz-Alvarez, Daniel Gonzalez-Gay, Raquel Ramos-Corral, Eva Marquez-Lietor, Nazaret Del Amo, Maria del Carmen Plata, Raquel Guillén-Santos, Ignacio Arribas, Fernando Cava-Valenciano

**Affiliations:** Central Laboratory BR Salud, Department of Clinical Chemistry, Infanta Sofia University Hospital, San Sebastian de los Reyes, Madrid, Spain; Department of Clinical Chemistry, Ramón y Cajal University Hospital, San Sebastian de los Reyes, Madrid, Spain

**Keywords:** bladder cancer, cutoff, CYFRA 21-1, lung cancer, non-small cell lung cancer (NSCLC), prognosis, tumor marker

## Abstract

**Objectives:**

Chosen cutoff for cytokeratin 19 fragment antigen (CYFRA 21-1) as a tumor biomarker considerably influences its diagnostic and prognostic usefulness. The aim of the present study is to determine an optimal cutoff value for diagnostic validity of CYFRA 21-1 by *Lumipulse* ^®^ technology in patients with suspected cancer and also to determine if CYFRA 21-1 levels provide prognostic value.

**Methods:**

A consecutive 284 patients suggestive of malignant disease from six hospitals of Madrid were enrolled in a retrospective design. Optimal CYFRA 21-1 cutoff value was obtained by receiver operating characteristic curve and Youden test. The diagnostic validity was evaluated according to sensitivity, specificity, predictive values and likelihood ratios. The prognostic value of CYFRA 21-1 was checked using multiple logistic regression. Thirty-two diagnostic cancers were confirmed.

**Results:**

The most optimal cutoff was 3.15 ng/mL. This cutoff showed a better specificity 93.63% (95% confidence interval [CI], 89.66–96.16), positive predictive value 60.98% (95% CI, 44.54–75.38) and positive likelihood ratio 12.65 (95% CI, 7.64–20.95) than the cutoff recommended by Fujirebio^®^ (1.8 ng/mL) (specificity: 73.71% [95% CI, 67.72–78.95], positive predictive value: 29.79% [95% CI, 21.02–40.23] and positive likelihood ratio 3.43 [95% CI, 2.71–4.35]), improving the current diagnostic accuracy. In multivariate analysis, elevated levels of CYFRA 21-1 (>3.15 ng/mL) was confirmed as an unfavorable prognostic factor.

**Conclusions:**

The best cutoff for CYFRA 21-1 obtained was 3.15 ng/mL in patients with suspected cancer. This new cutoff decreases the false positive rate and improves the diagnostic efficacy of CYFRA 21-1 as a tumor marker as well as its association with death events.

## Introduction

Cytokeratin 19 fragment antigen (CYFRA 21-1) belongs to a cytokeratin family, which is normally expressed in epithelial tissues and forms the epithelial cells’ filament cytoskeleton [1]. CYFRA 21-1 is useful as a tumor marker, especially for non-small cell lung cancer (NSCLC) along with carcinoembryonic antigen (CEA) and squamous cell carcinoma–associated antigen (SCC) [2], [3], but also for other epithelial tumors such as bladder cancer [4]. This elevation in tumors may be due to cell lysis, releasing cell contents to the blood, including CYFRA 21-1 by the action of proteases that degrade the cytokeratin filaments [5].

Cancer is the second cause of death in the world after ischemic heart disease, and lung cancer is the deadliest of all cancers [6]. Early diagnosis of lung cancer is critical, and although advances in imaging techniques have increased the diagnosis of lung cancer, most patients with NSCLC present advanced stage of the disease when the therapeutic options are already limited [7]. Early detection of disease progression during treatment is also crucial to save time and costs and to avoid unnecessary side effects from exposure to ineffective treatment [8]. Serum tumor markers can be used as a complementary utility to detect cancer, progression of tumor and treatment monitoring [9], [10], [11]. In fact, tumor markers are generally more useful to their prognostic value, and the diagnostic value is low, but certainly, in normal practice, clinicians often request tumor markers for this purpose, as a complementary use with clinical and other diagnostic tests. The diagnostic and prognostic efficacy of tumor markers depends greatly on the cutoff established by the laboratory and determines their specificity and sensitivity and therefore their effectiveness [12]. However, the CYFRA 21-1 cutoff is not clarified and there is no standardization between the different methods of analysis [13]. No optimal cutoff value has been determined by using *Lumipulse^®^* technology (Fujirebio^®^ Inc). Moreover, to establish an optimal cutoff level, it should be considered that high CYFRA 21-1 levels are also observed in other non-oncological diseases, such as renal insufficiency, liver disease and benign lung diseases (such as chronic obstructive pulmonary disease, infections), making cancer diagnosis more difficult.

CYFRA 21-1 cutoff by the *Lumipulse* ^®^ analyzer, recommended by the manufacturer, was set at 1.8 ng/mL (Fujirebio^®^ Inc). This cutoff value was set based on the data obtained from healthy adults. However, this cutoff has been shown too low for use in clinical practice in a population with suspected cancer, as the majority of these patients show high levels of CYFRA 21-1 even while suffering from benign diseases, thus confusing the clinicians. So in this case, it is more useful to increase the specificity of the test. Thus we carried out this study for assessment of CYFRA 21-1 cutoff by using *Lumipulse* ^®^ to improve the diagnostic efficacy of the marker and also to assess the prognostic information.

## Materials and methods

A retrospective observational study of serum CYFRA 21-1 levels was conducted in 284 consecutive patients suggestive of malignant disease between January and March 2018; 157 of them were men (55%) and 127 women (45%), between 25 and 94 years old (mean: 68 years).

Patients over 18 years with suspected cancer were also included. The suspicion of cancer was established on clinical criteria, such as fatigue, constitutional syndrome, persistent cough, localized pain and dyspnea. The definitive diagnosis of cancer was confirmed by histological examination of tumor tissue, which was considered the gold standard. Presence of renal disease was established as renal filtrate values (Chronic Kidney Disease Epidemiology Collaboration [CKD-EPI] formula) lower than 60 mL/min/1.73 m^2^ and liver disease as total bilirubin values higher than 4 mg/dL.

Cancer histological types were classified according to the 2015 World Health Organization recommendations of lung tumors [14] and bladder cancer [15].

Also, the same 284 patients were followed up for 12 months by reviewing their clinical histories coded to identify the relation of serum CYFRA 21-1 levels with the outcome (death) to determine the prognostic value.

Blood samples were collected in each hospital, centrifuged at 3500 *g* for 10 min, and the serum was stored at −40 °C. Serum CYFRA 21-1 was measured by a chemiluminescent enzyme immunoassay (CLEIA) in a *Lumipulse* ^®^ automated analyzer (Fujirebio^®^ Inc, Japan) in accordance with the manufacturer’s instructions. After measuring the hemolytic index quantitatively to all the samples, none of them was hemolyzed.

The assay was linear within the range between 0.5 and 100 ng/mL. The lower detection limit was 0.32 ng/mL and imprecision was less than 4.2% according to EP5-A2 protocol [16] of Clinical and Laboratory Standards Institute (CLSI) recommends a desirable variability of less than 5% [17].

The confidentiality of patients’ data has been maintained at all times by encoding their history numbers and reviewing their clinical histories anonymously. In addition, the patients verbally consented to the extraction of the sample and the analysis of CYFRA 21-1 to assess the diagnostic and prognostic value of the test along with their usual analytics, and so it did not involve the extraction of an additional sample. The project was authorized by the local investigation ethical commission. This study complies with the Helsinki Declaration agreement.

### Statistical analysis

Serum CYFRA 21-1 levels were expressed as median and interquartile range (IQR).

The optimal cutoff value for CYFRA 21-1 was obtained by receiver operating characteristic (ROC) curve and Youden test. The diagnostic efficacy was tested through sensitivity, specificity, positive predictive value (PPV), negative predictive value (NPV), positive likelihood ratio (PLR), negative likelihood ratio (NLR) and ROC curves with the area under the curve (AUC).

In the prognostic study, we have analyzed the associations between different parameters and mortality after transforming the values of each marker into values over and under the median and performing univariate analyses. The presence of confounders and interaction was analyzed in a stratified analysis. Finally, a multiple logistic regression model was built to identify variables independently associated with the outcome. Odds ratio (OR) was calculated with the 95% confidence interval (CI). The Hosmer–Lemeshow test was used to check the goodness of fit of the model.

Statistics are expressed with their 95% CIs and were performed with SPSS software (version 11.0; Chicago, USA). This study was performed according to the STARD statement [18] and REMARK guidelines [19].

## Results

After reviewing all clinical histories, a total of 32 definitive diagnoses of oncological disease were obtained from the histological analysis of biopsies (11%) and 252 (89%) had other diagnoses. From the total of 252 non-cancer group (other diagnoses), 31 patients were diagnosed with hepatic disease, 26 patients with renal insufficiency and 21 patients with chronic obstructive pulmonary disease (COPD). Different diagnoses are shown in Table 1.

**Table 1:** Final diagnoses and CYFRA 21-1 levels.

Oncological disease	n	Non-oncological disease	n
**Lung cancer**		**Hepatopathy**	31
Adenocarcinoma	9	**Renal insufficiency**	26
Squamous cell carcinoma	6	**Chronic obstructive pulmonary disease (COPD)**	21
Carcinoid tumor	2	**Urinary infection**	10
Mucoepidermoid carcinoma	1	**Respiratory infection**	9
Non-small cell carcinoma (unspecified)	6	**Heart failure**	9
**Bladder cancer**		**Gastritis**	5
Adenocarcinoma	3	**Emphysema**	4
**Metastasic cancer**			
Metastatic breast cancer	1	**Other benign diseases**	137
Metastatic colon cancer	1	(iron deficiency anemia, dyspnea, cholecystitis, without disease, diarrhea, anxiety, headache, polyps and constitutional syndrome)	
Metastatic gastric cancer	1		
Metastatic melanoma	1		
Metastatic tumor (unspecified origin)	1		
**Total, n**	**32**		**252**
CYFRA 21-1, median (IQR)	4.75 (3.89–7.70)	CYFRA 21-1, median (IQR)	1.20 (1.10–1.30)

CYFRA 21-1 levels: median (ng/mL) and IQR (interquartile range).

In the group of oncological diseases, most of the diagnosed tumors corresponded to lung cancer (75%), 10% to urothelial cancer and the rest (15%) to metastatic tumors, as shown in Table 1. From the total of cancer diagnosis, the majority of histologies were adenocarcinomas. The median of CYFRA 21-1 was significantly higher in the group of cancer (4.75 [3.89–7.70]) than in the non-cancer group (other diagnoses) (1.20 [1.10–1.30]). These results have been obtained using the Mann–Whitney U test (p<0.05) since the results followed a non-parametric distribution.

Using the current cutoff point (1.8 ng/mL) provided by Fujirebio^®^, a high false positive rate was observed (66 false positives: 23%) compared to only 16 true positives (5.7%), so a low specificity, PPV and PLR were observed in the statistical analysis (Table 2).

**Table 2:** CYFRA 21-1 diagnostic validity efficacy.

	Cutoff 1.8 ng/mL (95% CI)	Cutoff 3.15 ng/mL (95% CI)	Cutoff 3.15 ng/mL without interference^a^ (95% CI)
**Sensitivity**	**90.32%**	**80.65%**	**79.31%**
(95% CI)	(73.10–97.47)	(61.94–91.88)	(60.73–90.15)
**Specificity**	**73.71 %**	**93.63 %**	**95.54%**
(95% CI)	(67.72–78.95)	(89.66–96.16)	(92.12–97.35)
**Positive predictive value**	**29.79%**	**60.98%**	**69.70%**
(95% CI)	(21.02–40.23)	(44.54–75.38)	(52.98–80.32)
**Negative predictive value**	**98.40%**	**97.51%**	**97.27%**
(95% CI)	(95.03–99.59)	(94.40–98.98)	(93.87–98.11)
**Positive likelihood ratio**	**3.43**	**12.65**	**17.77**
(95% CI)	(2.71–4.35)	(7.64–20.95)	(11.23–24.75)
**Negative likelihood ratio**	**0.13**	**0.21**	**0.22**
(95% CI)	(0.04–0.39)	(0.10–0.42)	(0.11–0.43)
**Youden test**	**65.5%**	**71.8%**	**73.9%**

^a^Without interference: without renal and liver disease patients. Renal disease as renal filtrate values (CKD-EPI) < 60 mL/min/1.73 m^2^ or serum creatinine < 1.3 mg/dL and liver disease as bilirubin total > 4 mg/dL. Results are expressed in percentage (%), and range is expressed in 95% confidence interval (CI).

**Figure 1:**
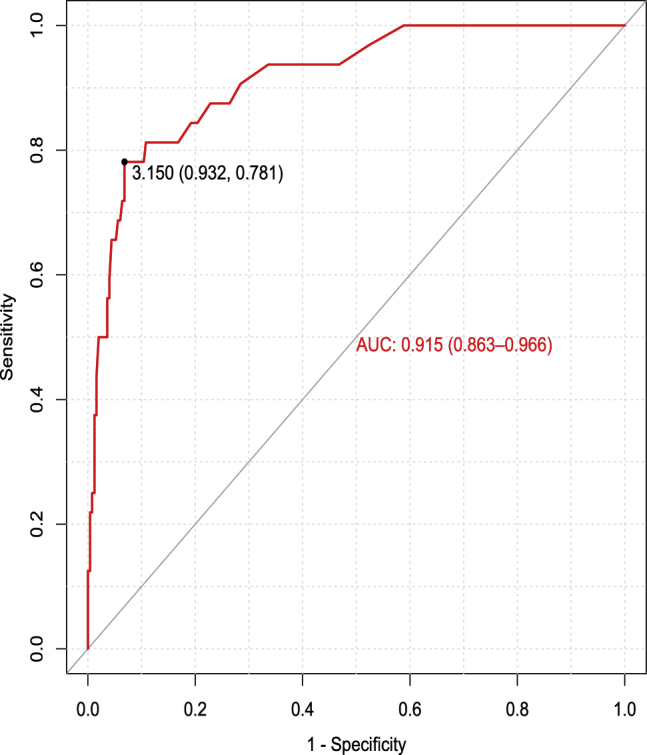
Graphical representation of different diagnostic efficiencies (sensitivity vs 1-specificity) according to different cutoff points.

Therefore, we considered obtaining the best CYFRA 21-1 cutoff point in our population using the Youden test. The Youden test obtained with the ROC curve (Figure 1) showed 3.15 ng/mL to be the optimal cutoff (71.8% Youden test) acquiring an AUC of 0.915 (95% CI, 0.863–0.966). This cutoff showed a better specificity of 93.63% (95% CI, 89.66–96.16), PPV of 60.98% (95% CI, 44.54–75.38) and PLR of 12.65 (95% CI, 7.64–20.95) than the cutoff currently recommended by Fujirebio^®^ Inc (1.8 ng/mL): Specificity: 73.71% (95% CI, 67.72–78.95), PPV: 29.79% (95% CI, 21.02–40.23) and PLR: 3.43 (95% CI, 2.71–4.35), improving the diagnostic accuracy of CYFRA 21-1 as a tumor marker.

If we would have considered as exclusion criteria those patients who had kidney or liver disease (considering the presence of renal disease as renal filtrate values, using CKD-EPI formula, lower than 60 mL/min/1.73 m^2^ and liver disease as total bilirubin values higher than 4 mg/dL) and, using the most optimal cutoff point (3.15 ng/mL), 29 patients belonging to these groups would not have been included and better results would have been obtained, especially in specificity, PPV and PLR (Table 2).

In the prognostic study, during the 12-month follow-up, two patients who belonged to benign diseases diagnoses group were lost, and thus 282 patients were finally included in this part of the study. A total of 32 death events were recorded during the next 12-month follow-up period, and six of them were diagnosed with non-oncological disease (19%). From the total of 32 deaths, 20 (62%) had high CYFRA 21-1 concentrations (1.8 ng/mL cutoff), whereas 12 patients (40%) had low CYFRA 21-1 concentrations.

Multivariate regression analysis showed that CYFRA 21-1 and age were independently associated with mortality (Table 3). The Hosmer–Lemeshow test was not significant (p=0.98) in validating the multivariate model.

**Table 3:** Multivariate logistic regression analysis.

Variable	p	OR adjusted	95% CI
**Age**	0.039	2.67	(1.55–9.12)
**CYFRA 21-1**	0.000	1.28	(1.11–1.75)

95% CI, confidence interval; OR, odds ratio.

## Discussion

Often symptoms of lung cancer are non-specific such as fatigue, dyspnea, pain or persistent cough and, overall in the first stages, are common to other diseases. In this way, it becomes necessary to include in addition to the medical history and symptoms a large number of tests to help diagnose it correctly, such as laboratory test, radiography, magnetic resonance imaging, computerized tomography (CT) [20], [21], bronchoscopy, biopsy, pulmonary function studies and eventually thoracentesis [22], [23]. Imaging techniques have unquestionable utility in tumor diagnosis and in monitoring the response to treatment, but against them, they have a high cost and are not exempt from causing some harm to patients [24]. The CT has a high sensitivity for the detection of pulmonary nodules, but the patient receives high radiation and most of the nodules are not carcinogenic. In a publication made in 2005 by Swensen et al. [25], nodules were detected in more than 70% of the patients and finally only 4% of them had lung cancer, in addition to subjecting them to a high dose of radiation. Therefore, an ideal method should be rapid, be of low cost and cause the least impact on patients. Serum tumor biomarkers could be a promising tool. Despite not having a high sensitivity, they have a good specificity, are cheaper and only require a blood test. In addition, it would increase the diagnostic capacity as a complement to imaging studies [26]. Nevertheless, there is no uniform criterion and the standardization of some tumor biomarkers is difficult [27]. Furthermore, their levels are also elevated in patients with benign lesions, although in these cases the increase is not as pronounced [28]. This is the case of CYFRA 21-1, which is present in healthy epithelial cells and malignant cells derived from the epithelium. For that reason, it is very important to establish an adequate cutoff point to help differentiate the effective and early diagnosis and prognosis of cancer from benign diseases. In the present study, the optimal cutoff obtained by using Lumipulse^®^ technology in our population was 3.15 ng/mL, which is similar to other cutoffs established by other authors for lung cancer diagnosis, although our population is not a healthy population and includes patients with suspected cancer (healthy and sick patients).

According to literature and Shuwei et al. [29] used an ROC curve to assess the cutoff value for CYFRA 21-1, establishing a cutoff value of 3.95 ng/mL and 3.20 ng/mL, respectively, which were consistent with previously published data. Liu et al. [30] also established a cutoff value of 3.3 ng/mL. Trapé et al. [31] conducted a research study of tumor markers in patients with symptoms of cancer, and they concluded that tumor markers improved their sensitivity in cancer diagnosis by using different cutoff points. There are many authors who support the idea of combining several tumor markers to improve the diagnostic efficacy in lung cancer, such as the combination of CYFRA 21-1 with CEA [32]. Even some authors suggest the highest diagnostic utility of combining a greater number of markers such as Molina et al. [33] who concluded that the diagnostic efficacy of lung cancer was improved by combining six tumor markers than using those same markers but individually. Liu et al. [34] recommended the combination of the same six tumor markers to distinguish the histological types of lung cancer.

Likelihood ratios (LRs), which are not influenced by the prevalence of the disease, are useful to assess the probability of each patient suffering the disease after the determination of the test (post-test probability). Applying Fagan’s nomogram [35] with our prevalence (11%) and using the cutoff of 1.8 ng/mL, the post-test probability of suffering cancer is 25% (PLR: 3.43) and the probability of discarding cancer decreases to 0.8% (using NLR: 0.13). Nevertheless, using the cutoff of 3.15 ng/mL, the post-test probability of suffering cancer rises to 65% (PLR: 12.65) and the probability of discarding cancer decreases to 1.8% (NLR: 0.21).

Because of the strong interference of renal and hepatic diseases in CYFRA 21-1 levels, we recommend to add a remark next to the CYFRA 21-1 value in the laboratory report that indicates “CYFRA 21-1 cutoff value recommended in patients with renal filtrate below 60 mL/min/1.73 m^2^ (or serum creatinine levels higher than 1.3 mg/dL) is higher; thus in these patients, CYFRA 21-1 levels cannot be interpreted.” Similarly, in the presence of liver disease, we recommend adding “CYFRA 21-1 cutoff value recommended in patients with liver disease is higher; therefore, in patients with total bilirubin values higher than 4 mg/dL, CYFRA 21-1 levels cannot be interpreted.”

In multivariate analysis, elevated (>3.15 ng/mL) levels of CYFRA 21-1 was confirmed as being an unfavorable prognostic factor. A meta-analysis published by Holdenrieder et al. [36] established that there was a high level of evidence for the clinical utility of CEA and CYFRA 21-1 to predict the treatment response in NSCLC. Kulpa et al. [2] also concluded that CYFRA 21-1 had a high prognostic value in the early stages of lung cancer. A meta-analysis published in 2017 [37] concluded that CYFRA 21-1 levels indicate higher stage cancer in NSCLC. According to Zhang et al. [38], CYFRA 21-1 individually had a greater prognostic value for lung cancer than CEA.

In conclusion, the best CYFRA 21-1 cutoff (performed on *Lumipulse^®^* analyzer) obtained in our population with clinical suspicion of cancer was 3.15 ng/mL. This value is higher than the cutoff provided by Fujirebio^®^ (1.8 ng/mL) and increases the PLR, decreasing the number of false positives and, therefore, unnecessary costs and inconvenience to the patient. This study has also demonstrated that elevated serum CYFRA 21-1 levels using the new cutoff (3.15 ng/mL) may be a useful non-invasive marker for identifying the risk of early death from NSCLC.

### Limitations

Cancer prevalence in Spanish population was 1.6% in 2017 (official data provided by the Spanish Statistical Office). However, the prevalence in this study was higher (11%) because tumor markers were only requested in patients with a clinical suspicion of cancer and the predictive values are overestimated. Nevertheless, the diagnostic efficacy should be tested with the LRs, which are not affected by disease prevalence. The results of this study should only be extrapolated to patients with suspected cancer.

The actual cutoff (1.8 ng/mL) leads to an excess of invasive diagnostic tests with an increase in costs and inconvenience to the patient, as side effects from radiation exposure.

In this study, we have not considered the influence of smoking status, sex or race in serum CYFRA 21-1 levels; nevertheless, some report that considering the influence of these factors does not present significant changes in CYFRA 21-1 levels [39].

Due to the strong influence of kidney disease, liver disease and benign pulmonary disease in the CYFRA 21-1 levels, it would be interesting to study the appropriate cutoff point to establish the diagnostic and prognostic value in patients with kidney diseases, liver diseases and benign pulmonary diseases using *Lumipulse^®^* methodology. In the present study, it has not been possible because the sample size of these patients was not enough to establish this cutoff, which would be interesting for future research.
